# The respiratory microbiota: new insights into pulmonary tuberculosis

**DOI:** 10.1186/s12879-019-3712-1

**Published:** 2019-01-25

**Authors:** Setegn Eshetie, Dick van Soolingen

**Affiliations:** 1 Departent of Medical Microbiology, School of Biomedical and Laboratory Sciences, College of Medicine and Health Sciences, University of Gondar, P.O.Box: 196, Gondar, Ethiopia; 2 National Institute for Public Health and the Environment (RIVM), 3720 BA Bilthoven, The Netherlands

**Keywords:** Respiratory system, Microbiota, Tuberculosis, Controls

## Abstract

**Background:**

Previous studies demonstrated that the diversity and composition of respiratory microbiota in TB patients were different from healthy individuals. Therefore, the aim of the present analysis was to estimate the relative proportion of respiratory microbiota at phylum and genus levels among TB cases and healthy controls.

**Methods:**

The PubMed and Google Scholar online databases were searched to retrieve relevant studies for the analysis. The statistical analysis was done using STATA version 11, pooled estimates are presented using graphs. The summary of findings in included studies is also presented in Table 1.

**Results:**

The phylum level analysis shows that the pooled proportions of *Firmicutes*, *Proteobacteria*, *Bacteroidetes*, *Actinobacteria*, and *Crenarchaeota* were determined among tuberculosis patients and healthy controls. In brief, *Firmicutes*, and *Proteobacteria* were the most abundant bacterial phyla in both TB cases and healthy controls, composing 39.9 and 22.7% in TB cases and 39.4 and 19.5% in healthy controls, respectively. The genus level analysis noted that *Streptococcus* (35.01%), *Neisseria* (27.1%), *Prevotella* (9.02%) and *Veillonella* (7.8%) were abundant in TB patients. The *Prevotella* (36.9%)*, Gammaproteobacteria* (22%), *Streptococcus* (19.2%) and *Haemophilus* (15.4%) were largely seen in healthy controls. Interestingly, Veillonella, *Rothia*, *Leuconostoc* were unique to TB cases, whereas *Lactobacillus*, and *Gammaproteobacteria, Haemophilus,* and *Actinobacillus* were identified only in healthy controls.

**Conclusion:**

The composition of the respiratory microbiota in TB patients and healthy controls were quite different. More deep sequencing studies are needed to explore the microbial variation in the respiratory system in connection with TB.

## Background

A microbiota is a group of microbial communities, namely bacteria, archaea, protists, fungi, and viruses, which have been described in various parts of human body. Commonly, the term microbiome is used to describe bacteria only, whereas the term mycobiome and virome are mostly used to describe fungal and viral populations, respectively. Understandably, the skin, gut, urogenital tract, upper respiratory tract and oral cavity are the common habitat of complex microbiomes [1, 2]. It was generally assumed that the lower respiratory system was free of microbial agents, but recent advancement demonstrated that these airways contain a complex variety of microbes, particularly abundant on the mucus layer and epithelial surfaces of the lower respiratory system. It is stated that the microbial diversity of the lung is determined by the immunity of the host, virulence of the microbes and the microbial content of the inhaling air [3–5].

To date, studies revealed several microbial agents in the lung of diseased and healthy controls. Notably, the most prominent bacterial phyla are *Bacteroides, Firmicutes, Proteobacteria, and Actinobacteria*, and the *Prevotella, Veillonella, but Streptococcus* and *Pseudomonas* were also commonly isolated bacterial genera in the respiratory samples of TB patients and controls. The pulmonary microbiota studies in human beings showed that type and diversity of microbes are affected by disease conditions, antibiotic therapy, environmental factors, and socio-demographic factors. Recent evidence showed that the composition of respiratory microbiomes in tuberculosis (TB) patients and healthy controls were different [2, 5, 6].

Though there are impressive global strategies in place, TB remains a public health threat, especially in resource-limited countries. According to the global reports, TB is considered as a significant cause of morbidity and mortality, worldwide [7, 8]. The classical diagnostic protocols for TB are mainly focused on the identification of *Mycobacterium tuberculosis* [2]. However, The development of TB may not only be determined by the primary agent of the disease but also governed by the interaction of local microbial communities and immunological factors [5, 9]. The new nucleic acid sequencing platforms are widely used to characterize the whole microbial diversity in the lung of TB patients, which could provide further insight into the pathogenesis of the disease [2, 10, 11]. To date, limited studies have been executed to investigate the respiratory microbiota in TB patients compared to healthy controls, but the results of the studies were quite conflicting and inconsistent [12, 13]. Therefore, the present study was aimed to summarize the recent findings in composition and diversity of microbes among TB patients and healthy individuals and to determine the pooled proportions of respiratory microbiomes at the phylum and genus levels.

## Methods

### Search strategy

A systematic literature search was conducted to identify relevant studies that assessed the composition and diversity of respiratory microbiota among TB and healthy controls. The PubMed, MEDLINE, Embase and Google Scholar online electronic databases were used to identify potentially relevant studies. The abstracts were retrieved by using the following key terms; “Lung microbiota” or “Respiratory microbiota” or “Sputum microbiota” and Tuberculosis or “Pulmonary tuberculosis” and “Healthy controls”. The relevant studies by title and abstract were further assessed for detail document evaluation. The studies with sufficient data that reported respiratory microbiome populations in both TB patients and healthy controls were included in the analysis. However, reviews and reports other than respiratory microbiota were excluded from the review. The systematic review and meta-analysis was conducted in accordance with Preferred Reporting Items for Systematic reviews and Meta-Analysis (PRISMA) guideline.

### Data synthesis and quality control

Study selection and extraction were carried out by the investigator (SE), and rechecking was made by the investigator (DS) to assess the quality of the included studies and whether the necessary parameters were considered for data extraction. The included studies were summarized by considering the following parameters; study name/reference, microbiome diversity at phylum and genus level in both TB cases and healthy controls, the method used for sequencing and sequencing region of 16S rRNA (Table 1). Cochrane collaboration’s risk of bias tool was used to assess the quality of the included studies. The Grading of Recommendations Assessment, Development and Evaluation (GRADE) system was used to evaluate the overall quality of the evidence. The quality was measured considering many domains, such as study design, precision, consistency, directness, and publication bias.

**Table 1:** Summary findings of the included studies, published from 2012 to 2016

Study	Respiratory microbiota				Method	Sequencing region of 16 s RNA
	TB patients		Healthy Controls			
	Phyla	Genera	Phyla	Genera		
Cheung et al	Proteobacteria*, Bacteroidetes*, Actinobacteria, Fusobacteria, Firmicutes	Neisseria* Streptococcus Prevotella*, Lactococcus, *Pseudomonas, Enterobacteria*	*Proteobacteria Bacteroidetes,* *ActinobacteriaFusobacteria* Firmicutes*	Streptococcus Neisseria Prevotella, Lactococcus*, Pseudomonas*, Enterobacter*	Roche/454	V1-V2
Wu et al	Firmicutes* Actinobacteria*, Spirochaetes*, Bacteroidetes, Fusobacteria, Cyanobacteria^−^*Thermi*^−^*Acidobacteria*^−^	*Prevotella*, *LeptotrichiaTreponema*, *Catonella* *Neisseria, Coprococcus* Streptococcus*,Gramulicatella*, Pseudomonas*, Haloplasma*,Bergeyella** Sharpea**	*Firmicutes*, *Actinobacteria Spirochaetes* Bacteroidetes* Fusobacteria* *Cyanobacteria* ^−^ *Thermi* ^−^ *Acidobacteria-*	Prevotella*, Leptotrichia*Treponema*Catonella* Coprococcus* *Streptococcus* *Neisseria, Gramulicatella Pseudomonas, Haloplasma*	Roche/454	V1-V2
Krishna et al	Firmicutes*, Actinobacteria* *Bacteriodes, Proteobacteria, Fusobacteria*	*Streptococcus, Neisseria, Veillonella* Unclassified-*Gammaproteobacteria* *Haemophilus, Actinobacillus* Rothia *, Leuconosto* Lactobacillus*, Corynebacterium*, Atopobium*, Bacillus*, Acinetobacter*	*Firmicutes, Actinobacteria* Bacteriodes, Proteobacteria* Fusobacteria*	*Streptococcus* *, Neisseria*, *Veillonella* Unclassified-*Gammaproteobacteria* Haemophilus, Actinobacillus*, Rothia *Leuconostoc, Lactobacillus* *Corynebacterium*, *Atopobium*, *Bacillus* Acinetobacter*	Ion Torrent	V6-V7
Cui et al	*Firmicutes, Bacteroidetes, ProteobacteriaCrenarchaeota Actinobacteria, Chlamydiae* *Chloroflexi, Cyanobacteria/Chloroplast* Deinococcus-Thermus*, Elusimicrobia, Euryarchaeota *SR1, Spirochaetes, Synergistetes, Tenericute* Fusobacteria, Aquificae** Caldiserica**, Gemmatimonadetes**, Lentisphaerae** Planctomycetes** Thermodesulfobacteria**, Verrucomicrobia**	Phenylobacterium* Stenotrophomona*, Cupriavidus* Sphingomonas*, Mobilicoccus* Brevundimonas* Brevibacillus* Diaphorobacter* Thermus**, Pelomonas** Methylobacterium**, Comamonas**, Lactobacillus** Thermobacillus**, Aur tidibacter**, Lapillicoccus** Devriesea**	*Firmicutes* Bacteroidetes*ProteobacteriaCrenarchaeota Actinobacteria *Chlamydiae, Chloroflexi, Cyanobacteria/Chloroplast* *Deinococcus-Thermus Elusimicrobia, Euryarchaeota* *SR1, Spirochaetes, Synergistetes, Tenericute* Fusobacteria*	*Phenylobacterium*, *Stenotrophomonas* *Cupriavidus* *SphingomonasMobilicoccusBrevundimonasBrevibacillus* *Diaphorobacter*	Roche/454	V3
Botero et al	Firmicutes, BacteroidetesProteobacteria, Actinobacteria*. Fusobacteria, Spirochaetes SR1, Gemmatimonadetes** Chloroflexi, Acidobacteria** *Tenericutes, Unclassified bacteria* *Ascomycota, Unclassified fungi*		*Firmicutes, BacteroidetesProteobacteria, Actinobacteria, Fusobacteria, Spirochaetes* *SR1, Chloroflexi* *Tenericutes, Unclassified bacteria, Ascomycota* *Unclassified fungi*		Roche/454	V1-V2

Key: * abundant microbiome either in TB cases or healthy controls, ** unique microbiome in TB cases

### Data analysis

A random effect model was used to determine pooled estimates. The pooled analysis was done for selected microbiomes (phylum, genus level) in TB patients and healthy controls. The statistical analysis was performed using STATA version 11, and the proportion of populations in the microbiome in patients and controls was presented in graphs. Firstly, the comparison was made with the pooled proportion of microbiome at the phylum level, and secondly, the overall estimate of microbial genera in both groups was determined.

## Findings

**Fig. 1:**
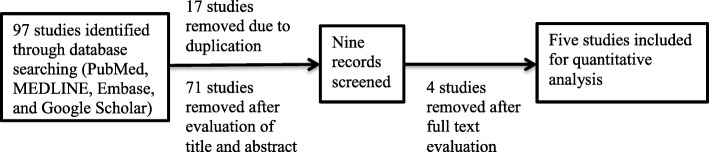
Preferred reporting items for systematic reviews and meta-analyses (PRISMA) flow-sheet

A total of 97 studies was retrieved by using the electronic database searches. Seventeen studies were immediately excluded because of duplication. The remaining studies were evaluated and 71 of them were ignored after reviewing the title and abstract. Finally, after full-text evaluation, five studies were included in the analysis [14–18] (Fig. 1). The included studies were published from the year 2012 to 2016. Majorly, the DNA sequencing was done using the Roche/454 method; only a single study used Ion Torrent for sequencing. With regard to the study setting, the four studies were done in China and the remaining one study originated from India. A sputum sample was almost invariably used as a material for analysis in TB cases, whereas various types of respiratory secretions were also employed to evaluate the diversity of microbial population in healthy participants.

As noted in Table 1, the distribution of the microbiome at both phylum and genus level were summarized as per TB cases and healthy controls. According to Cheung et al., five bacterial phyla were identified, notably, *Firmicutes*, *Proteobacteria, Bacteroidetes, Actinobacteria, andFusobacteria* were reported as the predominant bacterial phyla isolated in both TB cases and healthy controls. The phyla *Proteobacteria, and Bacteroidetes* were largely indicated in TB cases and the *Firmicutes* was abundant in healthy controls [14]. Likewise, the above-mentioned phyla were also noted by Krishna et al, but the relative abundance of the phyla in the two groups of participants was quite different from the aforementioned report by Cheung et al [16]. Moreover, a consistent finding was also reported by Botero et al as stated at the above Firmicutes, *Proteobacteria, Bacteroidetes*, *Actinobacteria*, and *Fusobacteria* were also the main microbial phyla in both TB patients and healthy controls. Typically, the study also showed that *Gemmatimonadetes* and *Acidobacteria* were found to be unique to TB patients. Besides, fungal microbiomes, such as *Ascomycota* and unclassified fungi were also demonstrated in samples of patients and controls [18].

Furthermore, additional bacterial phyla were also revealed by Wu et al*,* hence *Prevotella*, *Leptotrichia*, *Treponema*, *Catonella,* and *Coprococcus* were predominantly presented in healthy controls. In contrast, genera such as *Streptococcus*, *Gramulicatella,* and *Pseudomonas* were more abundant in TB patients than in healthy controls. Moreover, diverse groups of microbial phyla were explored in PTB and in healthy participants. In the identified microbiomes *Stenotrophomonas*, *Cupriavidus*, *Pseudomonas*, *Thermus*, *Sphingomonas*, *Methylobacterium*, *Diaphorobacter*, *Comamonas*, and *Mobilicoccus,* were exclusively presented in TB cases [15].

### Pooled analysis

**Fig. 2:**
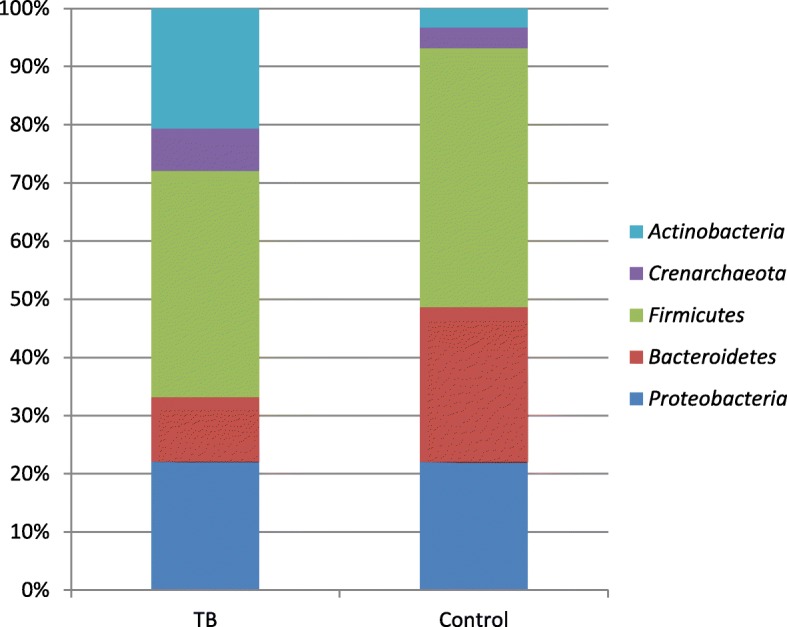
The relative abundance of microbial phyla among TB patients and health controls

In this analysis, the overall of proportions of microbial phyla and genera were estimated in both TB patients and healthy controls. However, the analysis was limited by the fact that the original studies reported extractable data only for some selected microbiomes. As presented in Fig. 2, the phylum level analysis shows that the pooled proportions of *Firmicutes*, *Proteobacteria*, *Bacteroidetes*, *Actinobacteria*, and *Crenarchaeota* were determined among patients and controls. Hence, *Firmicutes* and *Proteobacteria* were the most abundant bacterial phyla in both TB cases and healthy controls, composing 39.9 and 22.7% in TB cases and 39.4 and 19.5% in healthy controls, respectively. On the other hand, the relative abundance of *Actinobacteria* was seven times higher among TB cases compared to controls (21.2% versus 2.89%). Similarly, the pooled proportion of *Crenarchaeota* in TB cases was almost twice higher than among controls (7.5% versus 3.2%). In addition, statistically significant variation was also observed in presence of *Bacteroidetes*; it was observed in 23.5% of the healthy controls and in 11.4% of the TB cases.

**Fig. 3:**
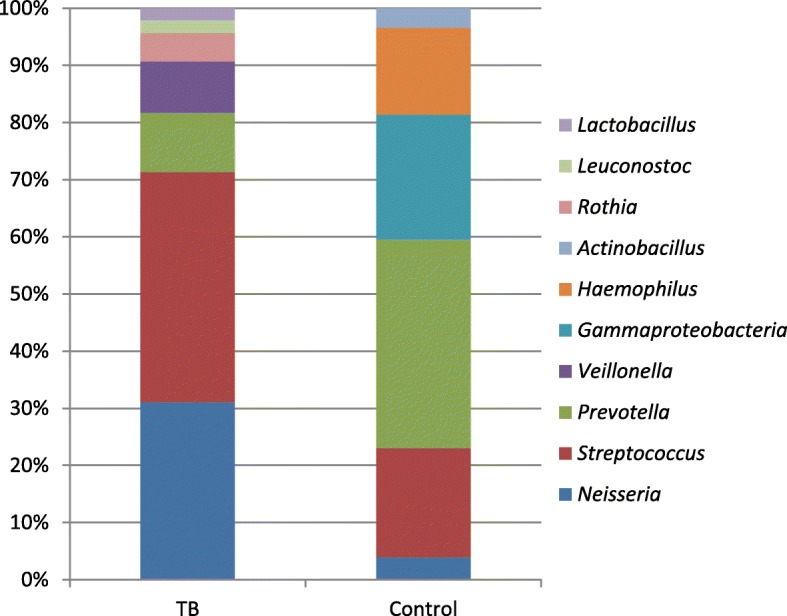
The relative abundance of microbial genera among TB patients and healthy controls

The differences in microbial abundance at the genus level between TB patients and healthy controls are presented in Fig. 3. *Streptococcus* (35.01%), *Neisseria* (27.1%), *Prevotella* (9.02%) and *Veillonella* (7.8%) were abundant in TB patients, whereas, *Prevotella* (36.9%)*, Gammaproteobacteria* (22%), *Streptococcus* (19.2%) and *Haemophilus* (15.4%) were also commonly observed in healthy controls. Comparatively, *Streptococcus* and *Neisseria* were more present in TB cases, and *Prevotella* was more prevalent in healthy controls. Most importantly, *Veillonella*, *Rothia*, *Leuconostoc*, and *Lactobacillus* were identified only in TB cases and detected in 7.8, 4.3, 2 and 1.8% of the patients, respectively. Besides, the genera *Gammaproteobacteria, Haemophilus,* and *Actinobacillus* were found to be unique to healthy controls.

## Discussion

The conventional culture technique remains the gold standard to investigate microbial infection, although the added value of molecular techniques in the diagnosis of infectious diseases is increasingly recognized*.* Considering the fact however that more than 90% microbial agents cannot be cultured with the current methods [2, 12], there it is obviously a reason to invest in molecular approaches. Recently, high throughput molecular techniques are becoming popular to investigate the composition and diversity of microbial flora. Notably, the 16 s rRNA sequencing platform is largely used for accurate identification of the microbiota of the human body. The whole genome and shotgun metagenomic sequencing have also been applied for comprehensive analysis of microorganisms [2, 19, 20]. The diversity and composition of the respiratory microbiota have been investigated in both normal, healthy individuals and diseased persons. It has been noted that the respiratory microbiota changes after infection by *Mycobacterium tuberculosis* [13, 21].

The main interest of characterizing microbial community is to determine whether a disease-specific condition is associated with a particular human microbiota [12, 21]. To the best of our knowledge, the role of the respiratory microbiota for the occurrence of latent or active TB remains unclear. Moreover, evidence showed that there is a unique microbial diversity among TB patients versus healthy controls, but the findings are so far controversial. For instance, according to Cheung et al *Bacteroidetes,* and *Firmicutes* were significantly observed in TB cases and healthy controls, respectively [14]. In contrast, Wu et al reported that *Firmicutes* was more presented in TB cases*,* whereas *Bacteriodetes* was largely seen in the samples from healthy controls [15]. Hence, the purpose of this study was to compile discoveries in the relation between the diversity of respiratory microbiota and TB. Besides, it was also meant to estimate the overall proportions of the predominant respiratory microbes among TB patients and healthy individuals.

The present study revealed that *Firmicutes, Proteobacteria*, *Bacteroidetes*, *Actinobacteria*, and *Crenarchaeota* were the major bacterial phyla demonstrated among TB cases and healthy controls. The *Firmicutes* and *Proteobacteria* were the most abundant bacterial phyla in both TB cases and healthy controls. However, statistically, significant differences were observed in the proportion of *Actinobacteria* and *Crenarchaeota* being more present in TB than healthy controls. In contrast*,* the *Bacteroidetes* was more common in healthy controls than in TB cases. Furthermore, the genus level analysis indicates that unique bacterial genera have been identified among TB cases and healthy controls. In brief; the pooled proportions of 10 bacterial genera have been estimated, hence *Streptococcus*, *Neisseria*, and *Prevotella* were commonly observed in both TB and healthy controls. On the other hand, *Veillonella*, *Rothia*, *Leuconostoc*, and *Lactobacillus* were found to be unique to TB patients. In contrast, the *Gammaproteobacteria, Haemophilus,* and *Actinobacillus* were observed only in healthy controls.

As mentioned before, few studies have been conducted to explore the microbial diversity in TB patients compared healthy individuals. Even more, the type and composition of respiratory microbiota were contrasting each other from one study to the other. Besides, the associations of viral and fungal microbes with tuberculosis were not sufficiently evaluated, since the fact that the previous studies have been carried out using 16S rRNA sequencing platform, which is not used to identify microbes other than bacteria. Therefore, it is suggested that more comprehensive analysis is needed, and detail investigation with the whole genome and shotgun sequencing should be considered to elucidate the microbial profile of the respiratory system accurately. Aside from the above, future research needs to be directed to pinpoint the role or impact of respiratory microbiota in TB occurrence and maturation of the local immunological aspects.

### Limitation of the study

First, the result of this study would be limited by the fact that pooled estimates of bacterial phyla and genera were only determined from studies that have reported extractable data on the relative abundance of the microbes. Second, few studies were included in the present analysis and therefore the power of the study might be compromised due to limited shreds of evidence. Moreover, the included studies have been conducted only in China and India; hence the findings of this study might not be generalizable at large.

## Conclusion

The present study has determined that the relative abundances of respiratory microbiota in TB patients were found to be different in comparison with healthy controls. Most importantly, some specific bacterial genera such as *Veillonella*, *Rothia*, *Leuconostoc*, and *Lactobacillus* were presented only in TB patients*.* The meticulous analysis using whole genome sequencing needed to be forwarded in order to have good knowledge of this aspect.
